# Prognostic significance of the Gustave Roussy immune (GRIm) score in cancer patients: a meta-analysis

**DOI:** 10.1080/07853890.2023.2236640

**Published:** 2023-10-18

**Authors:** Ke-Xin Sun, Ru-Qin Xu, Huan Rong, Hua-Yang Pang, Ting-Xiu Xiang

**Affiliations:** a Department of Oncology, The First Affiliated Hospital of Chongqing Medical University, Chongqing, China; b Chongqing Key Laboratory of Translational Research for Cancer Metastasis and Individualized Treatment, Chongqing University Cancer Hospital, Chongqing, China

**Keywords:** Cancers, Gustave Roussy immune score, survival outcomes, meta-analysis

## Abstract

**Background:**

The prognostic value of the Gustave Roussy immune (GRIm) score in cancer patients has been widely reported but remains inconsistent. The aim of this study is to systematically investigate the relationship between the GRIm score and survival outcomes in cancer patients.

**Methods:**

Relevant literature was identified using electronic databases including Web of Science, PubMed, and Embase from the inception to March 2023. The primary endpoints were long-term oncological outcomes. Subgroup analysis and sensitivity analysis were conducted during the meta-analysis.

**Results:**

Fifteen studies (20 cohorts) including 4997 cancer patients were enrolled. The combined results revealed that patients in the high GRIm group had a deteriorated overall survival (HR = 2.07 95%CI: 1.73–2.48; *p* < 0.0001; I^2^ = 62%) and progression-free survival (HR = 1.42; 95%CI: 1.22–1.66; *p* < 0.0001; I^2^ = 36%). The prognostic values of GRIm on overall survival and progression-free survival were observed across various tumour types and tumour stages. Sensitivity analysis supported the stability and reliability of the above results.

**Conclusion:**

Our evidence suggested that the GRIm score could be a valuable prognostic marker in cancer patients, which can be used by clinicians to stratify patients and formulate individualized treatment plans.

## 1. Introduction

With the increase in population size and the proportion of elderly people, cancer has become one of the leading causes of death globally [1]. Despite advances in surgery and medical treatment for cancer patients, the prognosis for these patients remains far from satisfactory [2]. Therefore, based on the estimated survival time of cancer patients, it is essential to develop individualized treatment strategies to improve their cure rate. At present, the anti-tumour therapy mainly relies on the traditional staging system. However, in clinical practice, the tumour-informed staging system alone is not able to support treatment selection as well as prognosis evaluation well [3,4]. Thereby, it is desirable to construct novel prognostic markers to guide more precise therapy of cancer patients.

The accumulating evidence suggests that host inflammation and nutritional status play an important role in the progression, drug response and long-term survival of cancer patients [5]. Based on this understanding, several inflammation/nutrition-related biomarkers have been constructed to predict the clinical outcomes of cancer patients, such as neutrophil to lymphocyte ratio (NLR) [6], prognostic nutrition index (PNI) [7] and systemic immune-inflammation index (SII) [8]. The Gustave Roussy immune (GRIm) score, which is calculated using serum lactate dehydrogenase (LDH), NLR and serum albumin, was first introduced by Bigot et al. [9] in 2017 as a prognostic assessment tool for cancer patients in the immunotherapy phase I clinical trials. Thereafter, the prognostic value of the GRIm score for predicting long-term outcomes was explored in different kinds of cancers [10,11]. Nonetheless, the prognostic value of GRIm score in cancer patients is in fact unclear due to conflicting results reported.

To the best of our knowledge, there has been no meta-analysis to investigate the prognostic value of GRIm scores in cancer patients. We, therefore, performed a pooled analysis based on the available evidence to systematically explore the relationship between GRIm score and survival outcomes in cancer patients.

## 2. Methods

### 2.1. Search strategy

The present meta-analysis was conducted according to the Preferred Reporting Items for Systematic Reviews and Meta-Analyses (PRISMA) guidelines to identify studies that evaluate the association of the GRIm score with survival outcomes in malignant patients. Relevant studies from Web of Science, PubMed and Embase were systematically examined up to 24 March 2023. The keyword ‘Gustave Roussy immune Score’ was applied to search related studies. During the search process, language restriction was not applied. Besides, the references of the enrolled studies were further scanned for extra reports. The search was performed by two investigators (KX-S and RQ-X) independently.

### 2.2. Study selection

Inclusion criteria: (1) Studies explored the association of the GRIm score and survival outcomes in cancer patients; (2) Hazard ratio (HR) with 95% confidence interval (CI) was directly reported or could be calculated; (3) Patients were divided into two groups based on the GRIm score (0–1 point: low risk group; 2–3 points: high risk group).

Exclusion criteria: (1) Studies were reported as letters, case reports, conferences or reviews; (2) Duplicated data.

### 2.3. Data extraction and quality assessment

Two reviewers (KX-S and RQ-X) independently conducted the data extraction and cross-checked all the extracted data. The extracted data included first author, publication year, country, study interval, study design, sample size, age, sex, cancer type, tumour stage, primary treatment, long-term survival data and follow-up time.

We performed the quality assessment of included studies according to the method by Lin et al. [12]. After careful assessment, a study could get a final score ranging from 0 to 9. Quality assessment was not used as an exclusion criterion for included studies.

### 2.4. Outcomes assessment

In the present study, the primary endpoints were survival outcomes including overall survival (OS), progression-free survival (PFS), disease-free survival (DFS) and cancer-specific survival (CSS). Since PFS, DFS and CSS share similar endpoints, they were analyzed together as one outcome, PFS, as previously suggested [13,14].

### 2.5. Statistical analysis

HRs with corresponding 95% CIs were used to analyze survival outcomes. For literature whose survival data were not directly reported, we extracted them from the survival curves according to the methods reported by Tierney et al. [15]. I^2^ statistics were applied to assess statistical heterogeneity among included studies. During pooled analysis, the random-effects model was performed to pool HRs. Subgroup analysis was performed to explore the source of heterogeneity and sensitivity analysis was applied to evaluate the stability of pooled results. Begg’s test was used to assess publication bias. P value <0.05 was considered statistically significant. Review Manager Software, version 5.3 (Cochrane, London, UK) and Stata, version 12.0 (Statacorp, College Station, TX) were used to perform all the statistical analyses.

## 3. Results

### 3.1. Study characteristics

**Figure 1.**
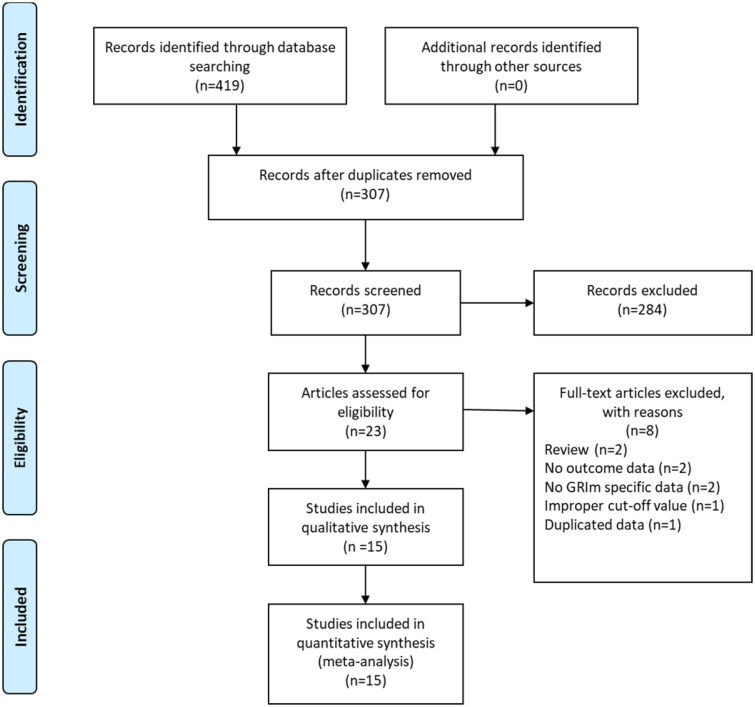
The PRISMA flowchart of study selection.

A total of 419 records were initially identified after searching these databases (Figure 1). By applying our inclusion criteria, 15 studies [9,11,16–28] with 20 cohorts were ultimately included in our meta-analysis. The basic information and clinical characteristics of these studies were shown in Table 1 and Table 2. Totally, 4997 patients from China, Japan, France, Italy, Canada, Austria and Turkey were included in this study. The included studies were published between 2017 and 2023, with a sample size ranging from 41 to 1579. The most common cancer type was lung cancer followed by gastrointestinal cancers. In terms of primary treatment, immune checkpoint inhibitors (ICIs) were performed in 9 cohorts, chemotherapy was performed in 5 cohorts, surgery was performed in 4 cohorts, target therapy was performed in 1 cohort and mixed treatment was performed in 1 study. The literature quality of these studies was good with a median score of 8 (range: 7–9, Figure 2 and Table S1).

**Figure 2.**
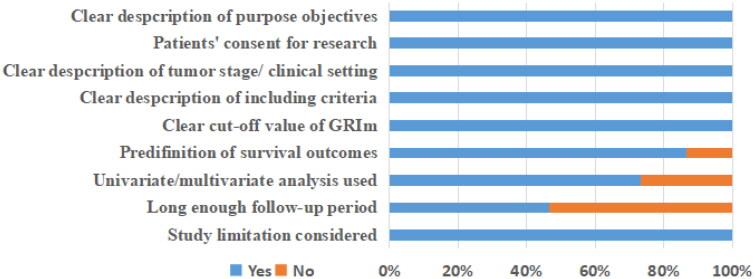
Quality assessment of included studies.

**Table 1.** The basic characteristics of included cohorts.

Author	Publication year	Country	Cancer type	Study design	study interval	Sample size	Age, years (median or mean)	Sex (male/female)
Basoglu	2022	Turkey	Pancreatic adenocarcinoma	R;S	2007–2019	138	62(range:36–78)	82/56
Bigot(1)	2017	France	Mixed	R;S	2012–2016	155	59(range:22–88)	83/72
Bigot(2)	2017	France	Mixed	P;S	2016	113	58(range:24–81)	79/34
Darazi	2020	France	Mixed	R;S	2015–2018	259	63(range:18–83)	169/90
Feng	2020	China	ESCC	R;S	2006–2010	372	59.3 ± 8.0	284/88
Kitadai	2019	Japan	NSCLC	R;S	2016–2019	41	NA	145/70
Lenci(1)	2021	Italy	NSCLC	R;M	2017–2020	135	71(range:44–91)	84/51
Lenci(2)	2021	Italy	NSCLC	R;S	2011–2017	87	73(range:36–88)	59/28
Li	2020	China	NSCLC	R;S	2014–2015	405	63.1 ± 7.6	256/149
Li(1)	2022	China	Hepatocellular carcinoma	R;S	2018–2019	181	NA	157/24
Li(2)	2022	China	Hepatocellular carcinoma	R;S	2019–2020	80	NA	45/35
Ma(1)	2023	Canada	Pancreatic adenocarcinoma	P;M	2015–2020	263	64(range:19–84)	157/106
Ma(2)	2023	Canada	Gastric and esophageal cancer	R;S	2007–2021	451	59(range:20–82)	308/143
Minami(1)	2019	Japan	NSCLC	R;S	2007–2018	185	68(IQR:62–74)	128/57
Minami(2)	2019	Japan	NSCLC	R;S	2007–2018	140	73(IQR:65–78)	55/85
Minami(3)	2019	Japan	NSCLC	R;S	2007–2018	115	71(IQR:66–75.5)	90/25
Minami	2020	Japan	SCLC	R;S	2007–2018	128	72.0(IQR:66.0–77.3)	101/27
Minichsdorfer	2022	Austria	Mixed	R;S	2015–2019	112	60(range:22–88)	74/40
Nakazawa	2022	Japan	Gastric cancer	R;M	2017–2018	58	66	45/13
Tian	2021	China	Colorectal cancer	R;S	NA	1579	58.11(range:21–85)	941/638

R: retrospective; P: prospective; S: single centre; M: multicentre; ESCC: oesophageal squamous cancer; NSCLC: non-small cell lung cancer; SCLC: small cell lung cancer; IQR: inter-quartile range; NA: not available.

**Table 2.** Clinicopathologic information of included cohorts.

Author	Publication year	Sample size (Low: High)	Primary treatment	TNM stage	Multivariate analysis	Survival outcomes	Median follow-up time, months
Basoglu	2022	138(111:27)	Surgery	Non-metastatic	Yes	OS	13.6 (range:7–31)
Bigot(1)	2017	155(114:41)	ICI	Metastatic	No	OS	6.5
Bigot(2)	2017	113(86:27)	ICI	Metastatic	No	OS	NA
Darazi	2020	259(150:109)	ICI	Mixed	No	OS	15
Feng	2020	372(314:58)	Surgery	Non-metastatic	Yes	CSS	NA
Kitadai	2019	41(NA)	ICI	Metastatic	No	OS;PFS	NA
Lenci(1)	2021	135(66:22)*	ICI	Mixed	No	OS;PFS	24
Lenci(2)	2021	87(41:45)*	CT	Mixed	No	OS;PFS	24
Li	2020	405(356:49)	Surgery	Non-metastatic	Yes	OS;DFS	50.0(range:12–66)
Li(1)	2022	181(NA)	ICI	Mixed	Yes	OS	17.7(IQR:12.6–22.9)
Li(2)	2022	80(NA)	ICI	Mixed	No	OS	10.1(IQR:8.2–14.8)
Ma(1)	2023	263(216:47)	CT	Mixed	Yes	OS	32.9
Ma(2)	2023	451(342:47)^a^	Mixed systemic therapy	Metastatic	Yes	OS	NA
Minami(1)	2019	185(139:46)	CT	Mixed	Yes	OS;PFS	NA
Minami(2)	2019	140(119:21)	TKI	Mixed	Yes	OS;PFS	NA
Minami(3)	2019	115(88:27)	CT	Mixed	Yes	OS;PFS	NA
Minami	2020	128(79:49)	CT	Mixed	Yes	OS;PFS	NA
Minichsdorfer	2022	112(83:29)	ICT	Metastatic	No	OS;PFS	NA
Nakazawa	2022	58(39:19)	ICT	Metastatic	No	OS;PFS	NA
Tian	2021	1579(1379:200)	Surgery	Mixed	Yes	OS;DFS	NA

ICI: immune checkpoint inhibitor; CT: chemotherapy; TKI: tyrosine kinase inhibitor; OS: overall survival; PFS: progression-free survival; DFS: disease-free survival; CSS: cancer-specific survival; IQR: inter-quartile range; NA: not available.

^a^Some cases without reported Gustave Roussy Immune Score.

**Table 3.** Subgroup analyses of overall survival between the high and low score groups.

		Cohorts, *n*	Patients, *n*	HR (95%CI)	*p* value	I^2^ (%)
Country	Total	19	4625	2.07(1.73–2.48)	<0.0001	62
	Asian	11	3050	1.76(1.45–2.13)	<0.0001	29
	Non-Asian	8	1575	2.54(1.94–3.34)	<0.0001	62
Study design	Prospective	2	376	5.98(3.11–11.51)	<0.0001	0
	Retrospective	17	4249	1.95(1.64–2.30)	0.002	56
Study center	Single center	16	4169	2.09(1.73–2.54)	<0.0001	64
	Multicenter	3	456	1.99(1.05–3.78)	0.03	63
Sample size	>150	8	3478	2.23(1.71–2.90)	<0.0001	71
	≤150	11	1147	1.94(1.49–2.52)	<0.0001	55
Cancer type	Gastrointestinal	7	2750	2.01(1.49–2.73)	<0.0001	70
	Lung	8	1236	1.68(1.34–2.11)	<0.0001	25
	Mixed	4	639	2.98(2.32–3.81)	<0.0001	13
Primary treatment	Surgery	3	2122	1.86(1.38–2.50)	<0.0001	0
	ICI	9	1134	2.27(1.68–3.07)	<0.0001	71
	CT	5	778	1.74(1.16–2.61)	0.007	67
	Others	2	591	2.53(1.99–3.22)	<0.0001	0
TNM stage	Non-metastatic	2	543	2.37(1.45–3.88)	0.006	0
	Metastatic	6	930	2.67(2.06–3.46)	<0.0001	40
	Mixed	11	3152	1.76(1.42–2.19)	<0.0001	59
Analysis method	Univariate	9	1040	2.30(1.75–3.01)	<0.0001	56
	Multivariate	10	3585	1.90(1.49–2.42)	<0.0001	65

### 3.2. Relationship between the GRIm and OS

**Figure 3.**
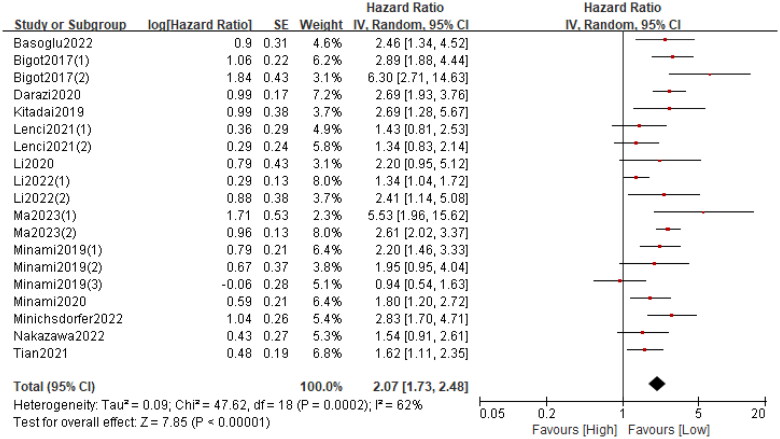
Forest plot Assessing the relationship between the GRIm and OS.

Nineteen cohorts involving 4625 patients described the relationship between the GRIm and OS. The pooled HR was 2.07 (95%CI: 1.73–2.48; *p* < 0.0001; I^2^ = 62%), which indicated that a high GRIm score was significantly associated with worse OS in patients with malignant tumour (Figure 3). Further­more, subgroup analyses based on country, study design, study centre, sample size, cancer type, primary treatment, TNM stage and analysis method were performed. As shown in Table 3 and Figure S1, the pooled results from all subgroup analyses revealed that patients in the high GRIm group had a substantially reduced OS when compared to those in the low GRIm group.

**Table 4.** Subgroup analyses of progression-free survival between the high and low score groups.

		Cohorts, *n*	Patients, *n*	HR (95%CI)	*P* value	I^2^ (%)
Country	Total	12	3357	1.42(1.22–1.66)	<0.0001	36
	Asian	9	3023	1.44(1.26–1.66)	<0.0001	0
	Non-Asian	3	334	1.38(0.80–2.40)	0.25	79
Study center	Single center	10	3164	1.47(1.21–1.78)	<0.0001	46
	Multicenter	2	193	1.31(1.02–1.69)	0.04	0
Sample size	>150	4	2541	1.54(1.26–1.88)	<0.0001	0
	≤150	8	816	1.38(1.10–1.73)	0.005	50
Cancer type	Gastrointestinal	3	2009	1.52(1.27–1.84)	<0.0001	0
	Lung	8	1236	1.24(1.04–1.49)	0.02	11
	Mixed	1	112	2.39(1.49–3.82)	0.0003	NA
Primary treatment	Surgery	3	2356	1.66(1.32–2.08)	<0.0001	0
	ICI	4	346	1.65(1.18–2.30)	0.004	52
	CT	4	515	1.10(0.90–1.34)	0.37	0
	Others	1	140	1.42(1.22–1.66)	0.05	NA
TNM stage	Non-metastatic	2	777	1.61(1.21–2.15)	0.001	0
	Metastatic	3	211	1.83(1.20–2.80)	0.005	60
	Mixed	7	2369	1.25(1.05–1.50)	0.01	18
Analysis method	Univariate	5	433	1.46(1.05–2.03)	0.02	66
	Multivariate	7	2924	1.43(1.22–1.68)	<0.0001	0

### 3.3. Relationship between the GRIm and PFS

**Figure 4.**
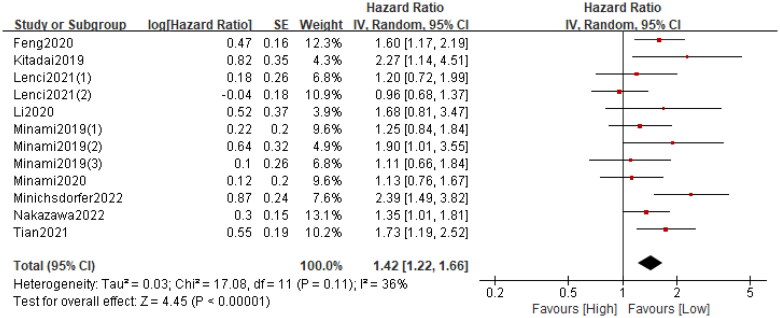
Forest plot Accessing the relationship between the GRIm and PFS.

A total of twelve cohorts consisting of 3357 patients reported PFS. The pooled HR was 1.44 (95%CI: 1.22–1.66; *p* < 0.0001; I^2^ = 36%), which suggested that patients in the high GRIm group had a significantly poorer PFS when compared with patients in the low GRIm group (Figure 4). Similarly, corresponding stratified analyses by country, study centre, sample size, cancer type, primary treatment, TNM stage and analysis method were performed. We found that in almost all subgroup analyses, patients in the high GRIm group has an inferior PFS, except for the pooled results from non-Asian population (*p* = 0.25) and those receiving chemotherapy (*p* = 0.37) (Table 4 and Figure S2).

### 3.4. Sensitivity analysis and publication bias

Sensitivity analysis was applied to assess the stability of the pooled results. By omitting one study at a time, we found that the pooled HRs with 95% CIs remained significant for both OS and PFS (Figure S3).

The Begg’s funnel plot was applied to assess potential publication bias. As shown in Figure S4, the funnel plots for OS and PFS were bilaterally symmetric and the P values were 0.326 and 0.449 for the Begg’s test, respectively.

## 4. Discussion

Uncontrolled inflammation and malnutrition are important features of cancer patients and can lead to poor response to medical treatment and worsening long-term outcomes in cancer patients [29,30]. Currently, a large amount of evidence has confirmed that peripheral blood-based parameters could be used as useful biomarkers reflecting inflammation and the nutrition status of cancer patients [31]. The minimally invasive retrieval, low cost and objectivity make complete blood count-based biomarkers highly attractive to clinicians, and more and more studies are focused on establishing biomarkers with clinical utility.

In this context, the GRIm score was developed by Bigot et al. [9] in 2017 as a potential prognostic assessment tool using three parameters (serum lactate dehydrogenase, NLR and serum albumin). After that, the GRIm score has been gradually applied to evaluate the prognosis of various malignancies owing to its easy availability and convenient calculation.

To determine the prognostic value of GRIm in cancer patients, we performed a comprehensive literature search and identified 15 studies with 4997 cancer patients. Through our meta-analyses, we found that the GRIm score was a negative prognostic predictor for OS and PFS in cancers. In addition, on examination of subgroup analyses based on cancer types, tumour stages, primary treatment, sample size and country, it can be seen that the pooled outcomes supported the efficacy of the GRIm score in the survival outcomes prediction. At the same time, the combined outcomes retained their significance on the sensitivity analyses, and no evidence of publication bias was observed through the Begg’s tests. Generally, all of these results suggested that the GRIm score played a significant predictive value in the prognosis of cancers.

The good discriminatory value of the GRIm score in various cancers could be attributed to its combined use of three important makers. Firstly, it has been shown that high plasma LDH concentration is beneficial for maintaining tumour anaerobic metabolism during tumour growth and metastasis, as well as for meeting tumour energy requirements in hypoxic conditions [32]. Meanwhile, LDH also exerts an inflammatory effect on the tumour microenvironment by activating IL-17 and IL-23, and inhibiting the activation of CD8+ T lymphocytes and natural killer cells, thereby enabling cancer cells to escape the immune response [33]. Moreover, a recent meta-analysis in­cluding 76 studies confirmed that high LDH plasma level was significantly associated with shorter PFS and OS in cancer patients [34]. Secondly, as a mature inflammation-related marker, NLR has been widely confirmed to predict short- and long-term adverse outcomes in haematologic and solid tumours [35]. The underlying mechanism is that neutrophils provide a favourable microenvironment for tumour cell proliferation and promote tumour cell progression and invasion [36]. Conversely, a reduction in lymphocyte numbers suppresses the immune response to cancer [37]. Finally, albumin is widely applied in clinical practice as a marker reflecting the nutritional status of cancer patients. Studies have shown that low serum albumin level delays wound healing, increases the risk of infection and reduces survival in patients with malignancy [38]. In addition, hypoalbuminemia is also closely associated with proinflammatory cytokine production and cell-mediated immunosuppression [39].

The present meta-analysis had several limitations. First, most of these included studies were designed to be retrospective, which may increase the risk of selection bias. Second, most included studies focused on lung cancer and digestive cancers, whereas other tumours like urinary tumours were lacking. Third, only aggregated data were included, and individualised data were not available for analysis.

## 5. Conclusions

In summary, our pooled results indicated that the GRIm score could be a valuable prognostic tool for cancer patients. Clinicians could use this useful index to stratify cancer patients and develop individual treatment strategies.

## Supplementary Material

Supplemental Material

## Data Availability

All data generated or analyzed during this study are included in this published article.

## References

[1] Sung H, Ferlay J, Siegel RL, et al. Global cancer statistics 2020: GLOBOCAN estimates of incidence and mortality worldwide for 36 cancers in 185 countries. CA Cancer J Clin. 2021;71(3):1–8. doi: 10.3322/caac.21660. 33538338

[2] Liu R, Zhao K, Wang K, et al. Prognostic value of nectin-4 in human cancers: a meta-analysis. Front Oncol. 2023;13:1081655. doi: 10.3389/fonc.2023.1081655. 36937394 PMC10020226

[3] Bando E, Ji X, Kattan MW, et al. Development and validation of a pretreatment nomogram to predict overall survival in gastric cancer. Cancer Med. 2020;9(16):5708–5718. doi: 10.1002/cam4.3225. 32588982 PMC7433838

[4] Ma L, Chen G, Wang D, et al. A nomogram to predict survival probability of gastric cancer patients undergoing radical surgery and adjuvant chemotherapy. Front Oncol. 2022;12:893998. doi: 10.3389/fonc.2022.893998. 35992865 PMC9389342

[5] Mantzorou M, Koutelidakis A, Theocharis S, et al. Clinical value of nutritional status in cancer: what is its impact and how it affects disease progression and prognosis? Nutr Cancer. 2017;69(8):1151–1176. doi: 10.1080/01635581.2017.1367947. 29083236

[6] Zhao X, Zhou Y, Liu B, et al. Preoperative neutrophil-lymphocyte ratio (NLR)-binding fibrinogen-albumin ratio (FAR) is superior to platelet-lymphocyte ratio (PLR)-binding fibrinogen-albumin ratio (FAR) and lymphocyte-monocyte (LMR)-binding fibrinogen-albumin ratio (FAR) as predictors of survival in surgical patients with colorectal adenocarcinoma. Med Sci Monit. 2023;29:e939442. doi: 10.12659/MSM.939442. 36992543 PMC10084709

[7] Dai M, Sun Q. Prognostic and clinicopathological significance of prognostic nutritional index (PNI) in patients with oral cancer: a meta-analysis. Aging. 2023;15(5):1615–1627. doi: 10.18632/aging.204576. 36897190 PMC10042682

[8] Wang Y, Ni Q. Prognostic and clinicopathological significance of systemic immune-inflammation index in cancer patients receiving immune checkpoint inhibitors: a meta-analysis. Ann Med. 2023;55(1):808–819. doi: 10.1080/07853890.2023.2181983. 36892953 PMC10795596

[9] Bigot F, Castanon E, Baldini C, et al. Prospective validation of a prognostic score for patients in immunotherapy phase I trials: the Gustave Roussy immune score (GRIm-Score). Eur J Cancer. 2017;84:212–218. doi: 10.1016/j.ejca.2017.07.027. 28826074

[10] Minami S, Ihara S, Ikuta S, et al. Gustave Roussy immune score and royal marsden hospital prognostic score are biomarkers of immune-checkpoint inhibitor for non-small cell lung cancer. World J Oncol. 2019;10(2):90–100. doi: 10.14740/wjon1193. 31068989 PMC6497012

[11] Feng JF, Wang L, Yang X, et al. Gustave Roussy immune score (GRIm-score) is a prognostic marker in patients with resectable esophageal squamous cell carcinoma. J Cancer. 2020;11(6):1334–1340. doi: 10.7150/jca.37898. 32047540 PMC6995395

[12] Lin Y, Liu Z, Qiu Y, et al. Clinical significance of plasma D-dimer and fibrinogen in digestive cancer: a systematic review and meta-analysis. Eur J Surg Oncol. 2018;44(10):1494–1503. doi: 10.1016/j.ejso.2018.07.052. 30100361

[13] Pang HY, Yan MH, Chen LH, et al. Detection of asymptomatic recurrence following curative surgery improves survival in patients with gastric cancer: a systematic review and meta-analysis. Front Oncol. 2022;12:1011683. doi: 10.3389/fonc.2022.1011683. 36387075 PMC9643694

[14] Yang XC, Liu H, Liu DC, et al. Prognostic value of pan-immune-inflammation value in colorectal cancer patients: a systematic review and meta-analysis. Front Oncol. 2022;12:1036890. doi: 10.3389/fonc.2022.1036890. 36620576 PMC9813847

[15] Tierney JF, Stewart LA, Ghersi D, et al. Practical methods for incorporating summary time-to-event data into meta-analysis. Trials. 2007;8:16. doi: 10.1186/1745-6215-8-16. 17555582 PMC1920534

[16] Al Darazi G, Martin E, Delord JP, et al. Improving patient selection for immuno-oncology phase 1 trials: external validation of six prognostic scores in a french cancer center. Int J Cancer. 2020;148(10):2502–2511. doi: 10.1002/ijc.33409. 33231298

[17] Basoglu T, Babacan NA, Ozturk FE, et al. Prognostic value of Gustave Roussy immune score in operable pancreatic adenocarcinoma. Indian J Cancer. 2022;60(2):179–184. doi: 10.4103/ijc.IJC_1049_20. 36861712

[18] Kitadai R, Okuma Y, Hakozaki T, et al. The efficacy of immune checkpoint inhibitors in advanced non-small-cell lung cancer with liver metastases. J Cancer Res Clin Oncol. 2020;146(3):777–785. doi: 10.1007/s00432-019-03104-w. 31828427 PMC11804625

[19] Lenci E, Cantini L, Pecci F, et al. The Gustave Roussy immune (GRIm)-score variation is an early-on-treatment biomarker of outcome in advanced non-small cell lung cancer (NSCLC) patients treated with first-line pembrolizumab. J Clin Med. 2021:10(5):1005. 10.3390/jcm10051005 PMC7958321 33801320

[20] Li SJ, Zhao L, Wang HY, et al. Gustave Roussy immune score based on a three-category risk assessment scale serves as a novel and effective prognostic indicator for surgically resectable early-stage non-small-cell lung cancer: a propensity score matching retrospective cohort study. Int J Surg. 2020;84:25–40. doi: 10.1016/j.ijsu.2020.10.015. 33086147

[21] Li Y, Pan Y, Lin X, et al. Development and validation of a prognostic score for hepatocellular carcinoma patients in immune checkpoint inhibitors therapies: the hepatocellular carcinoma modified Gustave Roussy immune score. Front Pharmacol. 2021;12:819985. doi: 10.3389/fphar.2021.819985. 35237150 PMC8883391

[22] Ma LX, Espin-Garcia O, Bach Y, et al. Comparison of four clinical prognostic scores in patients with advanced gastric and esophageal cancer. Oncologist. 2023;28(3):214–219. doi: 10.1093/oncolo/oyac235. 36378560 PMC10020804

[23] Ma LX, Wang Y, Espin-Garcia O, et al. Systemic inflammatory prognostic scores in advanced pancreatic adenocarcinoma. Br J Cancer. 2023;128(10):1916–1921. doi: 10.1038/s41416-023-02214-0. 36927977 PMC10147590

[24] Minami S, Ihara S, Komuta K. Gustave Roussy immune score is a prognostic factor for chemotherapy-naive pulmonary adenocarcinoma with wild-type epidermal growth factor receptor. World J Oncol. 2019;10(1):55–61. doi: 10.14740/wjon1184. 30834052 PMC6396777

[25] Minami S, Ihara S, Komuta K. Gustave Roussy immune score and royal marsden hospital prognostic score are prognostic markers for extensive disease of small cell lung cancer. World J Oncol. 2020;11(3):98–105. doi: 10.14740/wjon1275. 32494316 PMC7239571

[26] Minichsdorfer C, Gleiss A, Aretin MB, et al. Serum parameters as prognostic biomarkers in a real world cancer patient population treated with anti PD-1/PD-L1 therapy. Ann Med. 2022;54(1):1339–1349. doi: 10.1080/07853890.2022.2070660. 35535695 PMC9103267

[27] Nakazawa N, Sohda M, Ubukata Y, et al. Changes in the Gustave Roussy immune score as a powerful prognostic marker of the therapeutic sensitivity of nivolumab in advanced gastric cancer: a multicenter, retrospective study. Ann Surg Oncol. 2022;29(12):7400–7406. doi: 10.1245/s10434-022-12226-4. 35857197

[28] Tian S, Cao Y, Duan Y, et al. Gustave Roussy immune score as a novel prognostic scoring system for colorectal cancer patients: a propensity score matching analysis. Front Oncol. 2021;11:737283. doi: 10.3389/fonc.2021.737283. 34917499 PMC8669102

[29] Zhu J, Wang D, Liu C, et al. Development and validation of a new prognostic immune-inflammatory-nutritional score for predicting outcomes after curative resection for intrahepatic cholangiocarcinoma: a multicenter study. Front Immunol. 2023;14:1165510. doi: 10.3389/fimmu.2023.1165510. 37063918 PMC10102611

[30] Pang H, Zhang W, Liang X, et al. Prognostic score system using preoperative inflammatory, nutritional and tumor markers to predict prognosis for gastric cancer: a two-center cohort study. Adv Ther. 2021;38(9):4917–4934. doi: 10.1007/s12325-021-01870-z. 34379305

[31] Atasever Akkas E, Erdis E, Yucel B. Prognostic value of the systemic immune-inflammation index, systemic inflammation response index, and prognostic nutritional index in head and neck cancer. Eur Arch Otorhinolaryngol. 2023;280(8):3831–3833. doi: 10.1007/s00405-023-08053-2. 37289280

[32] Pelizzari G, Basile D, Zago S, et al. Lactate dehydrogenase (LDH) response to First-Line treatment predicts survival in metastatic breast cancer: first clues for a Cost-Effective and dynamic biomarker. Cancers. 2019;11(9):1243. doi: 10.3390/cancers11091243. PMC6770929 31450641

[33] Ding J, Karp JE, Emadi A. Elevated lactate dehydrogenase (LDH) can be a marker of immune suppression in cancer: interplay between hematologic and solid neoplastic clones and their microenvironments. Cancer Biomark. 2017;19(4):353–363. doi: 10.3233/CBM-160336. 28582845 PMC13020749

[34] Petrelli F, Cabiddu M, Coinu A, et al. Prognostic role of lactate dehydrogenase in solid tumors: a systematic review and meta-analysis of 76 studies. Acta Oncol. 2015;54(7):961–970. doi: 10.3109/0284186X.2015.1043026. 25984930

[35] Cupp MA, Cariolou M, Tzoulaki I, et al. Neutrophil to lymphocyte ratio and cancer prognosis: an umbrella review of systematic reviews and meta-analyses of observational studies. BMC Med. 2020;18(1):360. doi: 10.1186/s12916-020-01817-1. 33213430 PMC7678319

[36] Li Y, Xu T, Wang X, et al. The prognostic utility of preoperative neutrophil-to-lymphocyte ratio (NLR) in patients with colorectal liver metastasis: a systematic review and meta-analysis. Cancer Cell Int. 2023;23(1):39. doi: 10.1186/s12935-023-02876-z. 36855112 PMC9976405

[37] Melssen MM, Sheybani ND, Leick KM, et al. Barriers to immune cell infiltration in tumors. J Immunother Cancer. 2023;11(4):e006401. doi: 10.1136/jitc-2022-006401. PMC10124321 37072352

[38] Gupta D, Lis CG. Pretreatment serum albumin as a predictor of cancer survival: a systematic review of the epidemiological literature. Nutr J. 2010;9:69. doi: 10.1186/1475-2891-9-69. 21176210 PMC3019132

[39] Navaei-Alipour N, Mastali M, Ferns GA, et al. The effects of honey on pro- and anti-inflammatory cytokines: a narrative review. Phytother Res. 2021;35(7):3690–3701. doi: 10.1002/ptr.7066. 33751689

