# Supplementary material for: Prognostic significance of the Gustave Roussy immune (GRIm) score in cancer patients: a meta-analysis
Source: Ann Med. 2023 Oct 18;55(2):2236640. doi: 10.1080/07853890.2023.2236640 (PMC10586078; doi:10.1080/07853890.2023.2236640)
Supplement: Supplemental Material [file IANN_A_2236640_SM9894.docx]

Table S1. Quality assessment of included studies.

| **Items** | **Basoglu,2022** | **Bigot,2017** | **Darazi,2020** | **Feng,2020** | **Jian,2023** | **Kitadai,2019** | **Lenci,2021** | **Li,2020** | **Li,2022** | **Ma(1),2023** | **Ma(2),2023** | **Minami,2019** | **Minami,2020** | **Minichsdorfer,2022** | **Nakazawa,2022** | **Tian,2021** |
| --- | --- | --- | --- | --- | --- | --- | --- | --- | --- | --- | --- | --- | --- | --- | --- | --- |
| Study limitation considered | 1 | 1 | 1 | 1 | 1 | 1 | 1 | 1 | 1 | 1 | 1 | 1 | 1 | 1 | 1 | 1 |
| Long enough follow-up period | 1 | 0 | 0 | 1 | 0 | 0 | 0 | 1 | 0 | 1 | 0 | 0 | 0 | 1 | 1 | 1 |
| Univariate/multivariate analysis used | 1 | 1 | 1 | 1 | 1 | 1 | 0 | 1 | 1 | 1 | 0 | 0 | 1 | 1 | 0 | 1 |
| Predefinition of survival outcomes | 1 | 0 | 1 | 0 | 1 | 1 | 1 | 1 | 1 | 1 | 1 | 1 | 1 | 1 | 1 | 1 |
| Clear cut-off value of GRIm | 1 | 1 | 1 | 1 | 1 | 1 | 1 | 1 | 1 | 1 | 1 | 1 | 1 | 1 | 1 | 1 |
| Clear description of including criteria | 1 | 1 | 1 | 1 | 1 | 1 | 1 | 1 | 1 | 1 | 1 | 1 | 1 | 1 | 1 | 1 |
| Clear description of tumor stage/ clinical setting | 1 | 1 | 1 | 1 | 1 | 1 | 1 | 1 | 1 | 1 | 1 | 1 | 1 | 1 | 1 | 1 |
| Patients' consent for research | 1 | 1 | 1 | 1 | 1 | 1 | 1 | 1 | 1 | 1 | 1 | 1 | 1 | 1 | 1 | 1 |
| Clear description of purpose objectives | 1 | 1 | 1 | 1 | 1 | 1 | 1 | 1 | 1 | 1 | 1 | 1 | 1 | 1 | 1 | 1 |


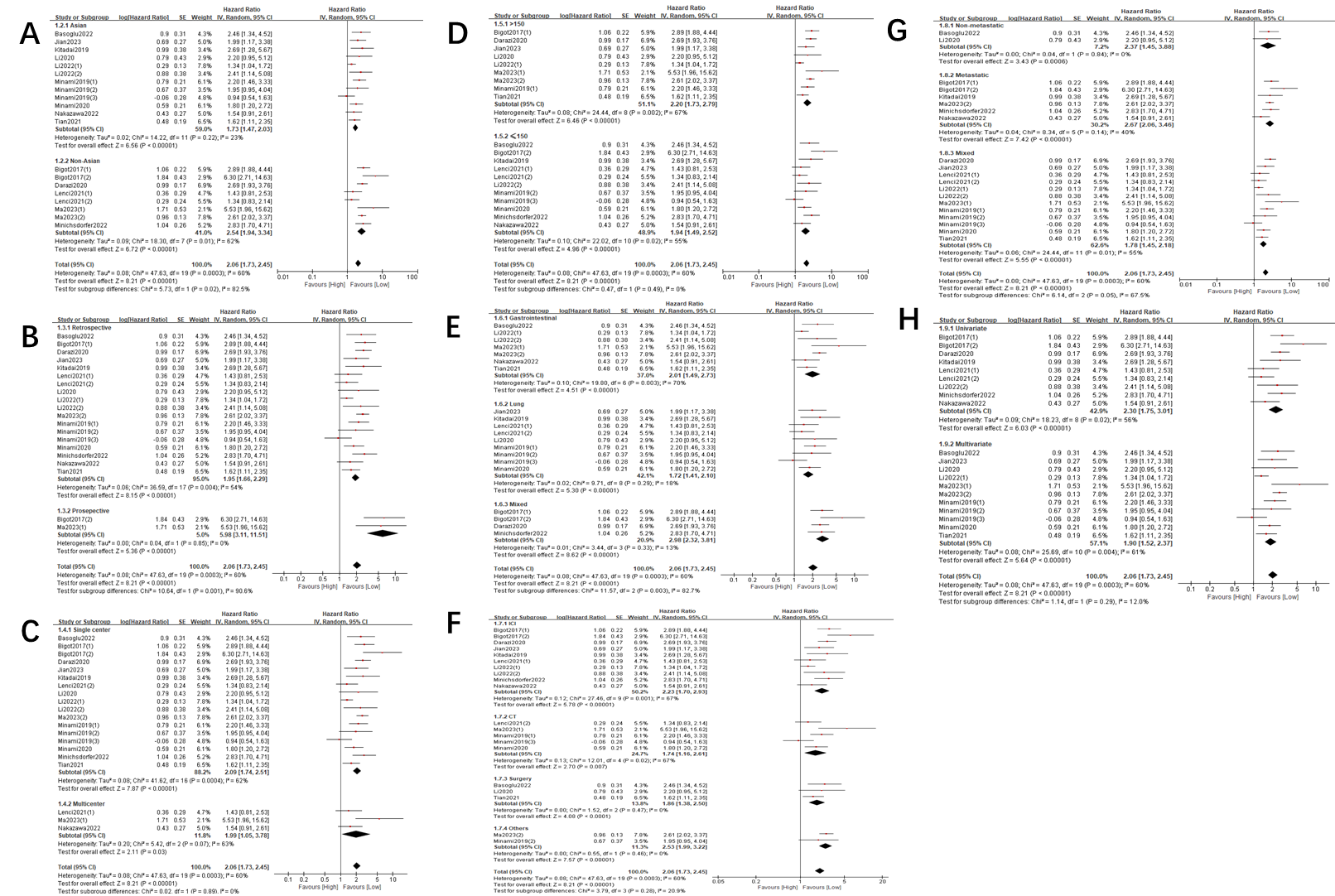


Figure S1. Forest plot of subgroup analyses assessing the relationship between the GRIm score and OS. A: Country (Asian vs. Non-Asian); B: Study design (Prospective vs. Retrospective); C: Study center (Single center vs. Multicenter); D: Sample size (>150 vs. ≤150); E: Cancer type (Gastrointestinal vs. Lung vs. Mixed); F: Primary treatment (ICI vs. CT vs. surgery vs. Others); G: TNM stage (Metastatic vs. Non-metastatic vs. Mixed); H: Analysis method (univariate vs. multivariate).


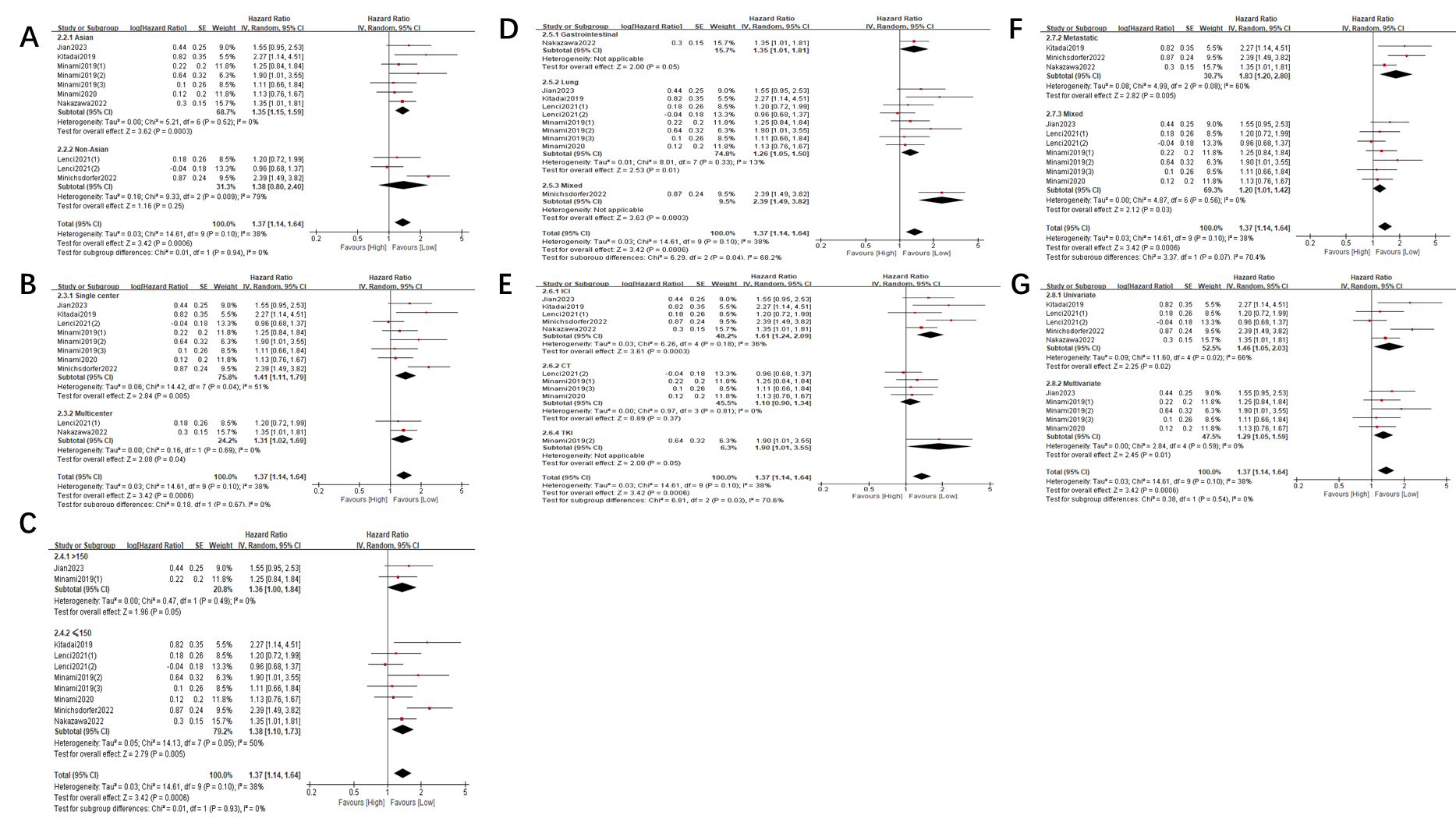


Figure S2. Forest plot of subgroup analyses assessing the relationship between the GRIm score and PFS. A: Country (Asian vs. Non-Asian); B: Study center (Single center vs. Multicenter); C: Sample size (>150 vs. ≤150); D: Cancer type (Gastrointestinal vs. Lung vs. Mixed); E: Primary treatment (ICI vs. CT vs. TKI); F: TNM stage (Metastatic vs. Mixed); G: Analysis method (univariate vs. multivariate).


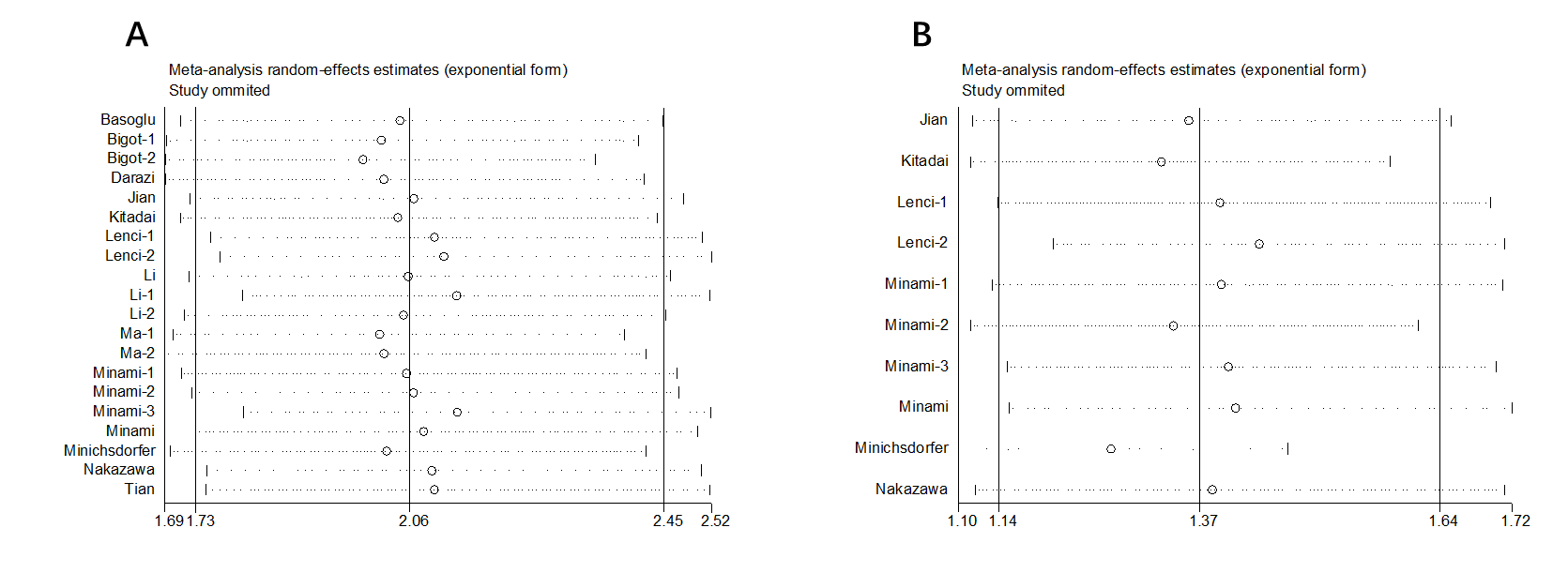


Figure S3. Sensitivity analyses assessing the relationship between the GRIm score and survival outcomes including OS (A) and PFS (B).


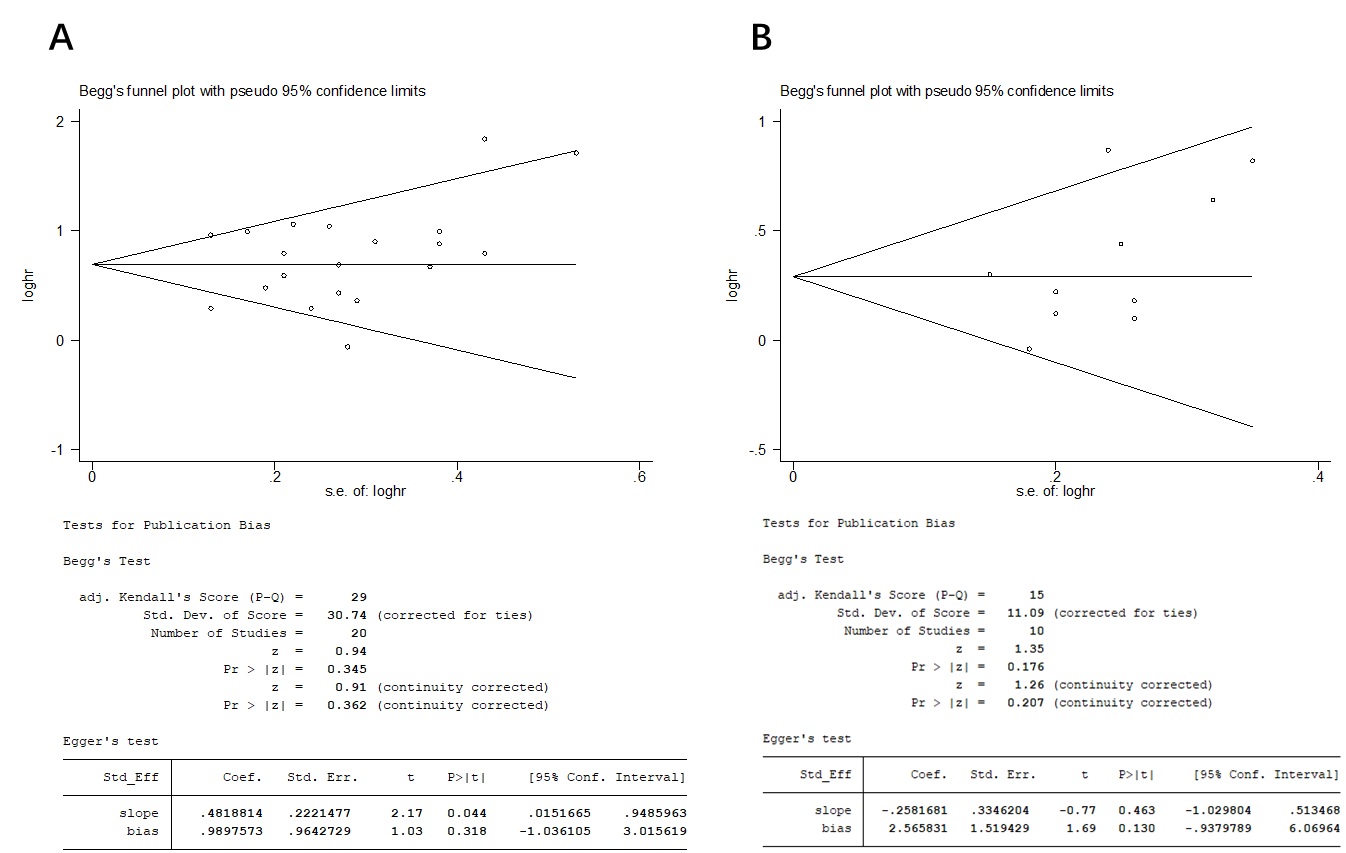


Figure S4. Begg’s funnel plots assessing publication bias between the GRIm score and survival outcomes including OS (A) and PFS (B). The Begg’s P values were 0.362 and 0.207, respectively.
